# Frequency of Hepatitis-B and C in patients undergoing cataract surgery in a tertiary care Centre 

**DOI:** 10.12669/pjms.314.6771

**Published:** 2015

**Authors:** Muhammad Ali Tahir, Alyscia Cheema, Saifullah Tareen

**Affiliations:** 1 Dr. Muhammad Ali Tahir, FCPS. Department of Ophthalmology, Jinnah Post Graduate Medical Centre, Karachi, Pakistan; 2 Dr. Alyscia Cheema, FCPS, FRCS. Department of Ophthalmology, Jinnah Post Graduate Medical Centre, Karachi, Pakistan; 3 Dr. Saifullah Tareen, FCPS. Department of Ophthalmology, Jinnah Post Graduate Medical Centre, Karachi, Pakistan

**Keywords:** Cataract Surgery, Hepatitis B, Hepatitis C

## Abstract

**Objective:**

To analyze the frequency of hepatitis B and C in patients undergoing cataract surgery.

**Methods:**

This descriptive study was conducted at department of Ophthalmology Jinnah Post Graduate Medical Centre. The duration of study was seven months from May 2013 to November 2013. After taking informed consent from the patient and hospital ethical committee all patients presenting with cataract and undergoing cataract surgery were evaluated for the existence of hepatitis C and B. Diagnosis of hepatitis C or B was made on the criteria that a patient must be positive for either Anti-HCV or HBsAg or both. Proformas were filled and data was collected and analysis was done. Pearson’s correlation coefficients were calculated to calculate the occurrence of hepatitis C and B in general population undergoing cataract surgery.

**Results:**

Six hundred and forty-eight patients were operated on for cataract surgery at Jinnah Post Graduate Medical Centre during the study period. Mean age of patients was 63 years, 300 (46.29%) were male and 348 (53.70%) female. Out of them 57 (8.79%) patients were carriers of either Hepatitis C or B. Hepatitis B accounted for 17 cases (2.62%) however Hepatitis C positive were 40 cases (6.17%). Nobody was simultaneously affected by both hepatitis C and B.

**Conclusion:**

Significant number of asymptomatic carriers of hepatitis C and B were found in preoperative cataract patients. It is recommended that preoperative screening of all cataract patients should be done so that asymptomatic carriers might not become a threat for spread of disease.

## INTRODUCTION

Hepatitis is a liver infection resulting in its inflammation that can progress leading to cirrhosis or cancer. Hepatitis is most commonly caused by viruses other causative factors are alcohol, poison and autoimmunity.^1^ Hepatitis B (HBV) and Hepatitis C (HCV) are of the viral types of hepatitis that are capable of causing acute and chronic form of hepatitis.^2^ According to a study hepatitis B is the cause of death in one million people each year. People who are affected by virus any time in their life time are estimated to be around two billion and carriers are thought to be around three fifty million.^3^ Prevalence of hepatitis C is 3% around the world.^4^ In 70 to 80% of cases it causes chronic liver diseases and later on liver malignancy.^5^ As far as Pakistani population is concerned the incidence of hepatitis B and C is on rise.^6^ According to some studies Pakistani population affected by HBV and HCV is around 10%^7^,^8^ and 4–10% respectively.^9^,^10^ Public and private sector hospitals are not routinely screening day care and admitted patients for hepatitis B and C therefore they are posing a serious threat to hospital staff and other patients.^11^

In most of the hospitals where unknown carriers of HCV and HBV are undergoing different procedures in which there is a chance of contact of percutaneous blood in the form of intravenous lines, incisions etc, surgeons, paramedical staff and other patients are at risk to get infected.^12^ According to some studies yearly percutaneous blood exposures among hospital staff in United States is estimated to be around five hundred thousand.^13^ A cataract is a clouding of the lens inside the eye which leads to a decrease in vision. It is the most common cause of blindness and is conventionally treated with surgery. Visual loss occurs because opacification of the lens obstructs light from passing and being focused on to the retina at the back of the eye.^14^ It is a matter of concern that surgeons and paramedical staff in Operation Theater are at great risk of occupational exposure.

Since the percentage of silent carriers of hepatitis B and C in Pakistan is still not very clear that is why this study was conducted to have at least an idea that with great amount of percutaneous exposures among hospital staff and health care providers they should be aware about the risk they are exposed to and take necessary actions beforehand. Due to this concern, study was carried out to evaluate the presence of hepatitis B and C infection in patients admitted for surgery at Jinnah Post Graduate Medical Centre Karachi.

## METHODS

This descriptive study was carried out at the department of Ophthalmology Jinnah Post Graduate Medical Centre. All patients of age 20 years or more, either gender, presenting for cataract surgery were evaluated for Hepatitis B and C infections. Patients less than 20 years of age and any other co morbidity like uveitis, glaucoma, retinal detachment were excluded. An informed written consent was obtained from all patients and information of subjects was kept confidential. This study was approved by the hospital ethical committee. Duration of study was seven months from May 2013 to November 2013. The intended sample size was 680 screened cataract patients however 648 patients agreed to participate in the study while 32 patients refused consent later on for various reasons. Thus the original sample size of 680 patients could not be completed.

The findings were recorded on proformas and analyzed through use of statistical tools of analysis which was carried out through statistical packaging soft ware system (SPSS) version 17. Screening technique used for the detection of surface antigen of hepatitis B and antibodies for hepatitis C was rapid chromatography immunoassay. Ethical considerations were taken into account.

## RESULTS

In this study 648 patients who were to be operated upon for cataract surgery were evaluated for the presence of HBV and HCV. Out of 648 patients males were 300 (46.29%) and females were 348 (53.70%). 63 years was the mean age. Age ranged from 20 to 75 years. Total 57 (8.8%) patients had either hepatitis B or C. No patient was found to be simultaneously affected with both hepatitis B and C. 17 (2.6%) patients were positive for hepatitis B and 40 (6.2%) for hepatitis C. Hepatitis B was found in 5 (1.67%) male and 12 (3.44%) female patients. Hepatitis C was predominant in females 22 (6.32%) while it was found in 18 (6.0%) male patients.34 out of 348 (9.7%) female patients had either hepatitis B or C however 23 out of 300 (7.67%) male patients had either hepatitis B or C. 277(92.3%) male patients and 314(90.2%) female patients had neither hepatitis B nor hepatitis C.

**Table-I:** Disease with gender.

Count					
Gender		No Disease	Hepatitis C Positive	Hepatitis B Positive	Total
	Male	277 (92.3%)	18 (6.0%)	5 (1.7%)	300
	Female	314 (90.2%)	22 (6.3%)	12 (3.5%)	348
Total	591 (91.2%)	40 (6.2%)	17 (2.6%)	648

## DISCUSSION

Prevalence of hepatitis B and C has reached an alarming level in Pakistan where large number of people are already infected with hepatitis B and C. According to some studies hepatitis B accounts for around 10%^7^,^8^ however hepatitis C has affected around 4-10%^9^-^10^ of general population. In rural areas of Pakistan the percentage of infected individuals is significantly higher than the above quoted figures.^7^-^9^ Very limited studies are available which cannot give clear scenario of prevalence of HBV and HCV at national level in particular among asymptomatic healthy individuals. Many studies have focused into small groups with some clinical indications therefore they cannot determine the overall prevalence in Pakistan.^15^

In our study we tried to screen those asymptomatic healthy individuals who came to us for cataract surgery. This study gave us the idea about silent carriers of hepatitis B and C. Although mean age of our study group was 63 years so it gave an idea of prevalence of silent carriers among elderly population. The transmission of virus is through the blood and secretions. Most common source of spread of these infections is through the use of unsterilized syringes or instruments especially dental instruments or unchecked blood transfusions, vertical transfer from mother to child and sexual abuse.^16^-^18^

In this study 2.62% patients had hepatitis B and 6.17% patients had hepatitis C, these figures are very close to the study conducted at Gadap area by Ali and his associates. According to them 5.1% patients were suffering from hepatitis C. HBs Ag carriers are around 10% in different areas of Pakistan.^15^ Contrary to that it was significantly less in our study and was about 2.62%. It may be possible that with in the span of sixteen years conditions have improved as people are getting more aware about spread of these blood borne diseases resulting in statistical improvement, secondarily our study patients came mostly from within the city so they may be having lesser incidence of carrier stage in comparison to the people residing in the rural areas.

In a study by Sheikh and his colleagues^19^ carrier state of HBs Ag was found to be 2.8% which was very much in accordance with that of our study of 2.62%. Weis and his coworkers^20^ reported 35% cases of HCV and 4% cases of HBV in their study of patients operated at John Hopkins. A study conducted in 2010 on different eye camps in Pakistan showed that 108 out of 437 patients were infected with Hepatitis B and Hepatitis C with a higher prevalence of the diseases in females with 60.18% (65/108) than in males with 39.81% (43/108)^21^ these figures show increased number of carriers as compared to our study in which out of 648 patients 57 patients were infected with hepatitis B or C but among those who were infected male and female percentages were very close to the above study with disease prevalence in females were 59.64% (34/57) and was higher than in males with 40.35% (23/57). This may be because our study is conducted in eye hospital located in Karachi where as above study was conducted in eye camps in rural areas where it is speculated that prevalence of hepatitis B and C is higher.

There were lot of short comings in our study as we did not included children less than 20 years of age in our study and according to some studies number of children developing Hepatitis B and C were on rise in Pakistan for past few years^22^ so our figures could be over or underrated. Secondly we did not take history of tattooing, significant sexual history, drug addiction history, and previous surgical and transfusion histories. These could have influenced the results. Further multi centre studies with greater number of patients involving all age groups should be done to tackle these issues.

## CONCLUSION

With such a rate of HBV and HCV as reported in our study, it suggests screening of all the patients who are selected for surgery. There is an urgent need for mass immunization against Hepatitis B. At the same time the print and electronic media should create public awareness about the methods of the spread of disease to prevent further transmission. It is the prime duty of doctors and paramedical staff to counsel the patients and do ethical practice. Department of health, Government of Pakistan should make laws and try to implement it within its resources that in all government run hospitals prior screening for transmissible diseases like Hepatitis B, Hepatitis C, AIDS etc should be mandatory before surgical procedures, and separate set of autoclave able instruments should be reserved for such patients to avoid spreading of these diseases at least from the hospitals where patients come for their treatment rather than getting transmissible diseases.
